# The motive of competition but not courtship positively correlates with self-reported use of aggressive humor: A critical test of the contests- vs. mate-choice hypotheses

**DOI:** 10.3389/fpsyg.2022.1056217

**Published:** 2023-01-11

**Authors:** Brent Duarte, Jinguang Zhang

**Affiliations:** ^1^Department of Communicology, University of Hawai‘i at Mānoa, Honolulu, HI, United States; ^2^School of Journalism and Communication, Sun Yat-sen University, Guangzhou, China; ^3^Center for Big Data and Public Communication, Guangzhou, China

**Keywords:** aggressive humor, verbal aggression, Contests Hypothesis, Mate-Choice Hypothesis, Dark-Triad personality

## Abstract

**Introduction:**

The use of aggressive humor (e.g., teasing, schadenfreude, and sarcasm) is a spiteful behavior because it inflicts costs on both others and the self. To explain the existence of this spiteful behavior, two hypotheses derived from sexual selection theory—namely Mate-Choice and Contests—posit that the use of aggressive humor helps one attract mates or repel competitors. Both hypotheses have merit, but extant data are unable to discriminate between them.

**Methods:**

We critically tested those two hypotheses with a survey study that measured 509 U.S. MTurkers’ self-reported tendencies to use aggressive (and other types of) humor, the motives to engage in competition and courtship, and the Dark-Triad personality traits. The final sample was *N* = 439.

**Results:**

We found that (1) the motive of competition but not courtship positively and significantly correlated with the self-reported tendency to use aggressive humor. (2) Subclinical psychopathy—a personality trait positively associated with competition—mediated the correlation between the motive of competition and self-reported use of aggressive humor. These results were held in both female and male respondents.

**Discussion:**

Our findings favored the Contests Hypothesis and helped reveal the psychological mechanism that generates the use of aggressive humor as a form of verbal aggression and spiteful behavior.

## 1. Introduction

At the 94th Academy Awards ceremony on March 27, 2022, comedian and host Chris Rock joked about actress Jada Pinkett Smith’s shaved head by quipping, “Jada, I love you. ‘G.I. Jane 2,’ can’t wait to see it. Al’ right?” While (some of) the audiences burst into laughter, Pinkett Smith apparently took offense and rolled her eyes. Will Smith—Jada’s husband—also laughed at the joke, but only for a brief moment before storming onto the stage and slapping a dumbfounded Rock across his face (France and Elam, 2022).

The “G.I. Jane 2” joke is an example of aggressive humor, which generates enjoyment and laughter by disparaging, ridiculing, and insulting others (Martin et al., 2003). From Joan Rivers and Don Rickles to Sarah Silverman and Chris Rock, and from the streets, night clubs, and all the way up to the White House Correspondents’ Dinners, jokes in the form of aggressive humor never cease to exist. From an evolutionary perspective, the wide practice of aggressive humor—especially amplified levels of aggressive humor such as schadenfreude and sarcasm (Torres-Marín et al., 2022)—needs an explanation: to the extent that the use of aggressive humor hurts others and the self (reviewed below), what produces this spiteful behavior?

The present research considers contests—the use of force or threats of force to exclude same-sex rivals from mating competition (Puts, 2010)—as a candidate mechanism, as did several other researchers. For example, Greengross and Miller (2008) proposed that aggressive humor may be used “to derogate, denigrate, insult, embarrass, and ostracize their sexual rivals” (p. 395). Kaufman et al. (2008) argued that “one can become the alpha male, or ‘top dog’ not just through brute force, but through humor—especially humorous derogation of sexual rivals” (p. 232). Cowan and Little (2013) argued that aggressive humor “could aid a user in intrasexual competition by allowing them to protect their reputation and self-image and could be considered a less risky strategy than physically aggressing against a competitor” (p. 161).

Despite that we are not the first to propose the Contests Hypothesis of the use of aggressive humor, there is to date no clear evidence for this hypothesis. As a result, it remains unclear whether contests or mate choice—a mechanism often invoked to explain human traits that show reliable sex differences—has played a more important role in selecting for the psychological mechanisms that produce the behavior of using aggressive humor. Clarifying this issue would help provide a more comprehensive characterization of the functions of the use of aggressive humor. This clarification, in turn, will advance our understanding of why such a spiteful behavior as using aggressive humor exists at all.

In what follows, we first review prior research on the costs of using aggressive humor. We then compare how the Mate-Choice and Contests Hypotheses explain the use of aggressive humor and evaluate how those two hypotheses fit with data from extant studies. We then describe a survey study that critically tests these two hypotheses.

### 1.1. Costs of using aggressive humor

The use of aggressive humor incurs on users themselves a series of social and physical costs. In terms of social costs, prior research found that often-users of aggressive humor tend to exhibit a plethora of personality traits considered socially undesirable. These include relatively low levels of agreeableness (Plessen et al., 2020), empathy (Navarro-Carrillo et al., 2020), and resilience (McCullars et al., 2021); and relatively high levels of anxiety (Dionigi et al., 2022), neuroticism (Jiang et al., 2020), and impulsivity (Geiger et al., 2019). Many of those traits—in particular low agreeableness and empathy, along with high neuroticism and impulsiveness—have been found to undermine one’s value as a friend and social exchange partner (Altmann, 2020; Thielmann et al., 2020).

To use aggressive humor also reduces one’s perceived value as a social partner. Zeigler-Hill et al. (2013) found that the use of aggressive humor correlated with higher levels of perceived aggressiveness, entitlement, and grandiosity and lower levels of perceived self-esteem, agreeableness, and emotional stability. Cann and Matson (2014) found that, compared with the use of affiliative and self-enhancing humor, the use of aggressive humor decreased one’s perceived sense of humor and social desirability. Cann et al. (2016) found that, compared to the use of affiliative humor, the use of aggressive humor caused one to be perceived as more arrogant, ruthless, and judgmental.

Finally, to use aggressive humor may end up hurting the self. Prior research found that using aggressive humor can induce aggressive responses from the audience (Berkowitz, 1970). As in the case of Chris Rock, trying to be funny in an aggressive manner invited a hard slap across his face.

### 1.2. A mate-choice explanation of the use of aggressive humor

The findings (and anecdotal evidence) reviewed above suggest that the use of aggressive behavior is a costly trait. A costly trait would be selected against by nature unless it has somehow promoted its carriers’ reproduction during the course of its evolution. Thus, despite its costs, the wide practice of aggressive humor suggests that the psychological mechanism that generates this behavior has been maintained by natural selection due to its positive effects on ancestral humans’ reproductive success. As one possibility, the use of aggressive humor might have increased its users’—especially male users’—attractiveness as potential mates.

According to sexual selection theory (Darwin, 1896), because men compared to women tend to have lower parental investment (Eibl-Eibesfeldt, 1989), men thus tend to have higher potential reproductive rates than women, resulting in a male-biased operational sex ratio (Clutton-Brock and Vincent, 1991). Thus, men compared to women would on average have to face more intense competition from same-sex rivals to gain access to members of the opposite sex for copulation (Puts, 2010). A strategy to gain such access would be to develop ornament traits to advertise one’s quality that women value (i.e., mate choice).

The ability to produce humor may be one such mental ornament trait. According to the Mental-Fitness Indicator Hypothesis (Miller, 2000), humor requires complex cognitive capacities (e.g., creativity) that tend to inversely correlate with the load of deleterious genetic mutations. It follows that humor production signals one’s verbal intelligence (Greengross and Miller, 2011; Christensen et al., 2018; but see, Driebe et al., 2021), mental health (Menéndez-Aller et al., 2020), and/or genetic quality. Thus, by mating with a funnier person, one is more likely to mate with a higher quality partner and produce higher quality offspring. In this process, men would generally be more motivated than women to produce humor (Greengross et al., 2020; Ross and Hall, 2020) because men typically face more intense intrasexual competition and thus more motivated to invest in courtship display, including humor production. In response, women should value men’s ability to produce humor (Bressler and Balshine, 2006; DiDonato et al., 2013; Hone et al., 2015; Tornquist and Chiappe, 2015), perceive romantic interest in men producing humor (Tornquist and Chiappe, 2020), and find men with relatively high humor capability to be attractive (Bressler and Balshine, 2006; Driebe et al., 2021).

Despite being insulting to others, aggressive humor intends to be funny and thus entails the creative use of language. Thus, following the Mental-Fitness Indicator Hypothesis, the ability to produce aggressive humor should also signal one’s verbal intelligence, mental health, and/or genetic quality. Thus, the Mental-Fitness Indicator Hypothesis would predict that (1) men should be more likely than women to produce aggressive humor to attract mates and (2) the use of aggressive humor should render men attractive to women in the mating context.

Consistent with the Mental-Fitness Indicator Hypothesis applied to explaining aggressive humor, men on average produce more aggressive humor than women (Martin et al., 2003; Dyck and Holtzman, 2013; Wu et al., 2016; McCullars et al., 2021). Also consistent with the hypothesis, Cowan and Little (2013) found that the use of aggressive humor increased men’s perceived attractiveness as short-term mates more so than as long-term mates, with a moderate effect size of Cohen’s *d* = 0.47. At the same time, though, the same authors found that the use of aggressive humor increased male users’ perceived dominance (i.e., the propensity or ability to inflict costs) more than it did to their perceived cooperativeness with a large effect size of Cohen’s *d* = 1.29. This latter result suggests that contests have played an important role in selecting for the use of aggressive humor. However, the relative impact between contests and mate choice remains unknown because Cowan and Little (2013) did not directly compare the effect of using aggressive humor on men’s perceived dominance and attractiveness. Further, Greengross and Miller (2008) found no evidence that the use of other-deprecating humor increased male or female participants’ perceived attractiveness as long- or short-term mates. Lastly, producing aggressive humor also unlikely signals mental health because it correlates with low levels of agreeableness, empathy, and resilience and high levels of anxiety, neuroticism, and impulsivity (see section “1.1 Costs of using aggressive humor”).

### 1.3. A contests explanation of the use of aggressive humor

With contests, men gain access to women as potential mates by repelling same-sex rivals (Puts, 2010). This mode of sexual selection has likely selected for weaponry traits in males, including men’s tendency to engage in physical aggression (Archer, 2009). Supporting this Contests Hypothesis of aggression, prior research found that status-competition and mating-motive primes increased men’s but not women’s aggression (Griskevicius et al., 2009; Ainsworth and Maner, 2014; Chen and Chang, 2015). While verbal aggression (i.e., using words to inflict psychological harm on others) (Buss and Perry, 1992) may not be as decisive as physical aggression in settling conflicts, the former often precedes the latter and can be violent as well (e.g., yelling) (Sell, 2011). Thus, verbal aggression should also have been subject to the contests mode of sexual selection (Archer, 2009).

Women also intrasexually compete (Campbell, 2013; Vaillancourt, 2013; Davis et al., 2018) because the fragility of human infants selects for paternal investment, which increases men’s importance to women’s reproduction (Geary, 2015). However, women are more likely to compete with verbal aggression than with physical aggression because the former generally incurs lower costs to women, who are responsible for performing most reproductive activities (Archer, 2009). As a result, the sex difference is less pronounced in verbal aggression than in physical aggression, albeit men are still more likely than women to verbally aggress (Buss and Perry, 1992).

Aggressive humor, which insults others by means of words, fits the definition of verbal aggression, and viewing aggressive humor as verbal aggression has several advantages. First, the Contests Hypothesis readily explains why men are more likely than women to use aggressive humor—men have generally faced more intense intrasexual competition than women and thus tend to be more aggressive (Archer, 2009; Puts, 2010). Second, the use of aggressive humor appears particularly effective in increasing men’s perceived dominance (Cowan and Little, 2013) because, if the Contests Hypothesis is correct, aggression—including verbal aggression and thus the use of aggressive humor—did *not* evolve to attract mates but to repel same-sex rivals. Consistent with this hypothesis, Brown et al. (2022) found that men and women tended to perceive more physically formidable (e.g., muscular) men to be more likely to use aggressive humor. Physical formidability is a major determinant of men’s ability to inflict physical costs on others and thus their competitiveness in contest competitions (Sell et al., 2012). Lastly, often-users of aggressive humor tend to exhibit higher levels of Machiavellianism and psychopathy (Dionigi et al., 2022) as well as spitefulness (Vrabel et al., 2017). This is likely because those traits facilitate aggressive contests (Carter et al., 2014; Goncalves and Campbell, 2014; Lyons et al., 2019).

### 1.4. The present study

In sum, the Mate-Choice and Contests Hypotheses each offer a functional explanation for the existence of the behavior of using aggressive humor, namely, to attract mates or repel rivals. There is evidence for both hypotheses (Cowan and Little, 2013; Brown et al., 2022), but—to our knowledge—none clearly differentiates the two. To fill this void, we in this research critically test whether the motives that correspond to contests and mate choice—that of competition (with same-sex others) and courtship—would correlate more strongly with the use of aggressive humor. Because the Contests Hypothesis is the focus of this research, we predict that the motive of competition would positively correlate with the use of aggressive humor (**Prediction 1**). However, if the Mate-Choice Hypothesis is correct, the motive of courtship would be a positive correlate.

#### 1.4.1. Proximate mechanisms

If competition motivates the use of aggressive humor as hypothesized, what are the proximate mechanisms? Machiavellianism (i.e., the predisposition to be manipulative and mistrusting) and psychopathy (i.e., the predisposition to be callous and reckless) (Furnham et al., 2013) are two candidates. We propose that the motive of competition should precede Machiavellianism and psychopathy because recent research suggests that personality traits are behavioral regularities generated by evolved psychological mechanisms to solve adaptive problems (Lukaszewski et al., 2020). Regardless of what generates Machiavellianism and psychopathy, those two personality traits—by being callous, manipulative, mistrusting, and reckless—are suitable for facilitating competition with the goal of repelling, if not eliminating same-sex rivals.

Thus, the chronic accessibility of the motive of competition should activate whatever psychological mechanisms that prescribe the behavioral patterns characteristic of high Machiavellianism and psychopathy (i.e., motive of competition → Machiavellianism and psychopathy), of which the use of aggressive humor is a tactic (i.e., Machiavellianism and psychopathy → use of aggressive humor). In other words, we predict that (a) Machiavellianism and (b) psychopathy would mediate the correlation between the motive of competition and use of aggressive humor (Prediction 2a and 2b). Consistent with Prediction 2, prior research found that the motive of competition, Machiavellianism, psychopathy, and use of aggressive humor significantly correlated with each other (Carter et al., 2014; Goncalves and Campbell, 2014; Lyons et al., 2019). However, no research has tested a mediation model that includes all these variables simultaneously.

We did not make predictions about an indirect effect through narcissism (i.e., competition → narcissism → use of aggressive humor) because it is difficult to determine *a priori* how feeling grandiose, entitled, and superior (Furnham et al., 2013) would help repel or eliminate mate rivals as being manipulative, mistrusting, callous, and reckless would. On the one hand, being relatively high on narcissism might reflect being confident, which may boost performance in competition. However, on the other, having “an exaggerated and inflated sense of their own importance” (Kjaervik and Bushman, 2021, p. 477) might cause one to underestimate the rival and lose the competition. Indeed, Lyons et al. (2019) found no evidence that the motive of competition correlated with narcissism. Thus, neither theory nor prior finding suggests a positive correlation between the motive of competition and narcissism.

Regarding the possible association between narcissism and the use of aggressive humor, Kjaervik and Bushman’s (2021) meta-analysis found a significant positive correlation between being narcissistic and engaging in all forms of aggression. Research also found that narcissism—especially its grandiose dimension—negatively correlates with empathy (Urbonaviciute and Hepper, 2020) and agreeableness (Zajenkowski and Szymaniak, 2021). However, none of the three studies cited above appeared to have controlled for the potential confounding effects of Machiavellianism and psychopathy. To what extent narcissism would correlate with aggression after its covariances with the remaining two “dark” personality traits is removed remains unknown. Given these considerations, we decided to leave the possible correlation between narcissism and the use of aggressive humor as a research question.

Finally, we did not make predictions on the use of the other types of humor (e.g., affiliative, self-enhancing, and self-defeating) because no theory or prior evidence compelled us to do so. We included them as statistical controls because the use of the four types of humor might reflect a common psychological mechanism underlying “humor production,” the potential confounding effect of which needs to be ruled out for a stringent test of our hypothesis.

## 2. Materials and methods

### 2.1. Respondents

A sample of *N* = 509 US adults from the Amazon Mechanical Turk (MTurk) participated in the study for a small payment. As recommended (Kennedy et al., 2020), we required that all MTurk workers have a HIT^1^ approval rate greater than 95% to ensure data quality. Nine workers participated in our study twice, and we dropped their second-time response. An additional 61 workers self-identified as homosexual or bisexual, and we excluded their data following prior research on human intrasexual competition (Ainsworth and Maner, 2014; Chen and Chang, 2015; Davis et al., 2018) because this study focused on men’s and women’s motive of competing against same-sex others for access to members of the opposite sex. We were thus left with *N* = 439 for the final sample, which consisted of 50.8% males and 71.3% whites and had a median age of 37 years (ranging from 18 to 73 years). The protocol of this research was approved by the University of Hawai‘i at Mānoa Institutional Review Board (#2020-00192).

### 2.2. Procedure and measures

After providing informed consent, respondents were first asked to provide demographic information, including age, sex, ethnicity, sexual orientation, and relationship status. Doing so enabled us to assign respondents to questions that assessed their motives of mating competition and courtship depending on their self-reported sex and sexual orientation.

#### 2.2.1. Self-reported use of humor

Next, respondents were asked to complete the 32-item Humor Style Questionnaire (HSQ) (Martin et al., 2003) that measured their self-reported tendency to use affiliative (e.g., “I enjoy making people laugh”), self-enhancing (e.g., “If I am feeling depressed, I can usually cheer myself up with humor”), aggressive (e.g., “If someone makes a mistake, I will often tease them about it”), and self-defeating (e.g., “I let people laugh at me or make fun at my expense more than I should”) humor (1 strongly disagree, 7 strongly agree). The internal consistency of all four subscales were satisfactory by convention: Cronbach’s α = 0.88 for affiliative humor, α = 0.78 for self-enhancing humor, α = 0.88 for aggressive humor, and α = 0.85 for self-defeating humor. Higher values indicate stronger self-reported tendencies to use a particular type of humor.

#### 2.2.2. Motives of competition and courtship

Next, we adapted Chen and Chang’s (2015) 26-item scale to measure respondents’ motives of competition and courtship. Example items included “I would compete with other men for the woman I like” (i.e., competition for males) and “I would do everything to attract the woman I like” (i.e., courtship for males), as well as “I would compete with other women for the man I like” (i.e., competition for females) and “I would do everything to attract the man I like” (i.e., courtship for females). Both subscales (i.e., competition and courtship) showed excellent internal consistency (Cronbach’s α = 0.95 and 0.91), with higher values indicating stronger motives of competition or courtship.

#### 2.2.3. Dark-triad personalities

Finally, respondents were asked to complete the 27-item Short Dark Triad scale (SD3) (Jones and Paulhus, 2014) that measured Machiavellianism (Cronbach’s α = 0.88), narcissism (α = 0.84), and sub-clinical psychopathy (α = 0.84). Higher values indicate higher levels of each personality trait.

## 3. Results

### 3.1. Descriptive statistics, data preparation, and analytic strategy

Table 1 presents descriptive statistics. We applied logarithmic and square root methods to correct the skews of self-reported use of affiliative (before: skew = −0.52; after: skew = 0.32, *SE*s = 0.11) and self-enhancing (before: skew = −0.55; after: skew = 0.03, *SE*s = 0.11) humor. We then *Z*-transformed all variables so that the magnitude of raw regression coefficients indexes the size of correlations.

**TABLE 1:** Descriptive statistics and intercorrelations.

Variable	*M*	*SD*	1	2	3	4	5	6	7	8
1. Aggressive	3.39	1.09								
2. Affiliative	5.20	1.18	0.13**							
3. Self-enhancing	4.79	1.16	0.18**	0.53**						
4. Self-defeating	3.62	1.21	0.42**	0.06	0.18**					
5. Competition	2.87	1.43	0.37**	-0.19**	0.06	0.43**				
6. Courtship	4.78	1.16	0.12*	0.07	0.19**	0.34**	0.51**			
7. Machiavellianism	3.04	0.89	0.49**	-0.09*	0.01	0.33**	0.61**	0.36**		
8. Psychopathy	2.12	0.83	0.54**	-0.20**	0.06	0.45**	0.70**	0.32**	0.66**	
9. Narcissism	2.57	0.85	0.30**	0.05	0.18**	0.25**	0.57**	0.38**	0.53**	0.58**

*p < 0.05. **p < 0.01.

Including age as a covariate did not alter statistical conclusions (see Supplementary Table 1), and we thus dropped age to keep our models parsimonious. We used the full sample to test Prediction 1 and 2 before stratifying analyses by respondents’ sex to explore potential sex differences in the main findings. See Supplementary Table 2 for the sex differences in the self-reported use of all four types of humor. All path models—including mediation analyses—were estimated using maximum likelihood with standard errors robust to non-normality in R (R Core Team, 2020) with the “lavaan” package (Rosseel, 2012).

### 3.2. Test of prediction 1

**FIGURE 1:**
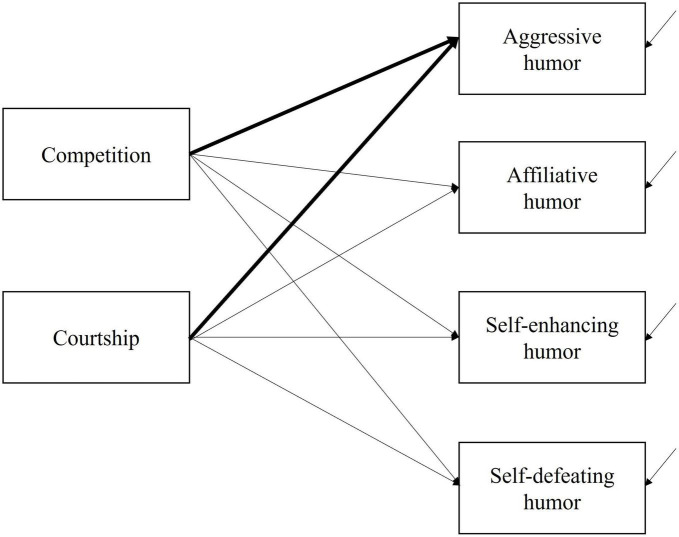
Regressing self-reported use of aggressive and other types of humor of motives of competition and courtship in a path model. Thicker arrow lines indicate focal paths. Arrow lines pointing toward the four types of humor represent residual terms. The covariances of the residual terms were controlled for in model estimation but omitted in the figure for clarity of presentation.

Prediction 1 stated that the motive of competition would positively correlate with self-reported use of aggressive humor. To test this prediction, we ran a path model specified in Figure 1 below and summarized the results in Table 2. Fit indices are not available because the model is saturated.

**TABLE 2:** Results of path models predicting self-reported use of humor from competition and courtship.

Parameter	*b*	*SE*	*Z*	*p*
**Predicting aggressive humor**				
Competition	0.42	0.04	9.79	<0.001
Courtship	−0.09	0.05	−1.65	0.10
**Predicting affiliative humor**				
Competition	−0.30	0.05	−5.85	<0.001
Courtship	0.20	0.06	3.36	0.001
**Predicting self-enhancing humor**				
Competition	−0.08	0.05	−1.54	0.13
Courtship	0.21	0.06	3.32	0.001
**Predicting self-defeating humor**				
Competition	0.35	0.05	7.50	<0.001
Courtship	0.17	0.05	3.12	0.002

Confirming Prediction 1 derived from the Contests Hypothesis, the motive of competition positively and significantly predicted self-reported use of aggressive humor. Inconsistent with the Mate-Choice Hypothesis, there is no evidence that the motive of courtship predicted self-reported use of aggressive humor.

Table 2 also revealed that the motives of competition and courtship significantly correlated with self-reported use of the other three types of humor. However, it was only with self-reported use of aggressive humor that the motive of competition emerged as a positive predictor. For self-reported use of affiliative and self-enhancing humor, the motive of competition emerged as a negative predictor and the motive of courtship, a positive predictor. For self-reported use of self-defeating humor, the motives of competition and courtship both emerged as positive predictors.

As mentioned in section “1.3 A contests explanation of the use of aggressive humor,” there is a possibility that the results for Prediction 1 (summarized in Table 2) reflected a common psychological mechanism that produces maladaptive humor or humor altogether. Indeed, the intercorrelations among the self-reported use of the four types of humor were substantial, particularly between the self-reported use of aggressive and self-defeating humor (Table 1). To rule out that possibility, we first ran an ordinary-least-square regression model predicting self-reported use of aggressive humor from the motive of competition, the motive of courtship, and self-reported use of the remaining three types of humor. Results were similar as those reported in Table 2: the motive of competition was a significant positive predictor of self-reported use of aggressive humor [*b* = 0.36, *t*(433) = 6.91, *p* < 0.001] and the motive of courtship was a significant negative predictor [*b* = −0.18, *t* = −3.76, *p* < 0.001].

We then repeated the above analysis except, this time, predicting self-reported use of self-defeating humor (i.e., another type of maladaptive humor per prior research) with self-reported use of aggressive humor as a covariate. Also similar to the findings reported in Table 2, the motives of competition [*b* = 0.23, *t*(433) = 4.32, *p* < 0.001] and courtship [*b* = 0.18, *t*(433) = 3.70, *p* < 0.001] both emerged as significant positive predictors. Thus, the finding that the motives of competition and courtship differentially correlated with self-reported use of aggressive humor was not confounded by a “common core” of (maladaptive) humor production.

### 3.3. Test of prediction 2

**FIGURE 2:**
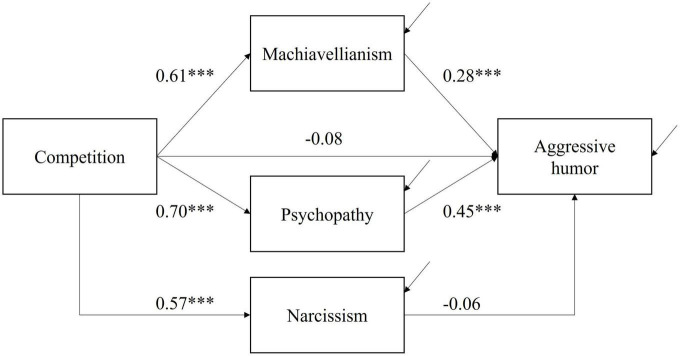
Indirect effects of Machiavellianism and psychopathy. Arrow lines pointing toward Machiavellianism, psychopathy, narcissism, and aggressive humor represent residual terms. The covariances of the residual terms of the mediators (i.e., Machiavellianism, psychopathy, and narcissism) were controlled for in model estimation but omitted in the figure for clarity of presentation. ***p < 0.001.

Prediction 2 stated that (a) Machiavellianism and (b) psychopathy would mediate the correlation between the motive of competition and self-reported use of aggressive humor. Given the results related to the test of Prediction 1, we specified the path model as depicted in Figure 2. We included narcissism to control for the covariances among the three “dark” personality traits and to explore whether narcissism would predict use of aggressive humor. Fit indices are not available because the model is saturated.

As predicted, the indirect effect of “competition → Machiavellianism → aggressive humor” was estimated *b* = 0.17 and significant, with the 95% bootstrapped CI estimated [0.09, 0.24]. The indirect effect of “competition → psychopathy → aggressive humor” was estimated *b* = 0.31 and also significant, with the 95% CI estimated [0.21, 0.41]. The indirect effect of “competition → narcissism → aggressive humor” was estimated *b* = −0.03, with the 95% CI estimated [−0.10, 0.03]. Finally, the direct correlation between competition and aggressive humor was estimated *b* = −0.08 and was no longer significant (*p* = 0.25). These findings support Prediction 2a and 2b.

Because the indirect effect through narcissism was not significant, we next explored whether Machiavellianism or psychopathy accounted for the direct correlation between the motive of competition and self-reported use of aggressive humor. We first ran a path model only with Machiavellianism as the mediator (i.e., competition → Machiavellianism → aggressive humor), and the direct correlation between the motive of competition and self-reported use of aggressive humor was significant, *b* = 0.11 (*SE* = 0.05), *Z* = 2.24, *p* = 0.03. In comparison, when we replaced Machiavellianism with psychopathy as the mediator, the direct correlation between the motive of competition and self-reported use of aggressive humor reduced to practically zero, *b* = −0.01 (*SE* = 0.06), *Z* = 0.20, *p* = 0.84. Thus, psychopathy appeared to be the main mechanism driving the correlation between the motive of competition and self-reported use of aggressive humor. These results remained largely the same when we included the use of affiliative, self-enhancing, and self-defeating humor as covariates (Supplementary Table 3).

**FIGURE 3:**
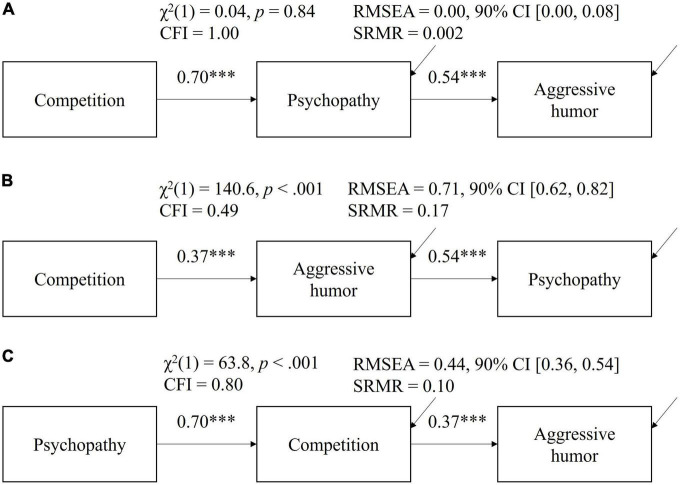
Comparing the fit of three competing indirect-effect models with model **(A)** as our proposed model and models **(B,C)** as alternative models. Arrow lines pointing toward mediator- and outcome variables represent residual terms. Larger *p*-values of the chi-square test, larger CFI values, smaller RMSEA values, and smaller SRMR values indicate better model fit. ***p < 0.001.

Given these findings, we fitted three additional path models (Figures 3A–C) to explore how (1) the motive of competition, (2) psychopathy, and (3) self-reported use of aggressive humor correlated with each other.

As seen from Figure 3, our hypothesized model fitted the data the best by all key indices, suggesting that the effect flowed from the motive of competition to psychopathy and then to self-reported use of aggressive humor.

### 3.4. Did prediction 1 and 2 hold in both sexes?

To address this question, we stratified the path models depicted in Figures 1–3 by respondents’ sex. Regarding Prediction 1, the motive of competition remained a positive and significant predictor of aggressive humor in both female and male respondents (*b*s = 0.48 and 0.36, *p*s < 0.001). At the same time, the correlation between courtship and aggressive humor remained negative in both female and male respondents (*b*s = −0.15 and −0.18, *p*s = 0.06 and 0.01). See Supplementary Tables 4, 5 for details.

Regarding Prediction 2, the indirect effect of Machiavellianism remained significant in both female (*b* = 0.13, 95% CI [0.03, 0.23]) and male (*b* = 0.19, 95% CI [0.08, 0.29]) respondents, as did that of psychopathy (*b* = 0.33, 95% CI [0.21, 0.44]) for female respondents and (*b* = 0.21, 95% CI [0.07, 0.35]) for male respondents (see Supplementary Tables 6, 7). For female respondents, as with the full sample, controlling for psychopathy but not Machiavellianism dropped the correlation between the motive of competition and self-reported use of aggressive humor to non-significant. However, for male respondents, controlling for either Machiavellianism or psychopathy reduced the direct correlation between the motive of competition and self-reported use of aggressive humor to non-significant (see Supplementary Tables 8, 9). Overall, both Prediction 1 and 2 were held in both sexes. Lastly, the indirect effect model “competition psychopathy aggressive humor” remained the best fitted model in both female and male respondents. See Supplementary Figures 1, 2.

## 4. Discussion

This research likely provided the first critical test of the Contests and Mate-Choice hypotheses of the use of aggressive humor. With a US adult sample, we found that the motive of competition but not that of courtship positively and significantly correlated with self-reported use of aggressive humor. Subclinical psychopathy—and to a lesser extent, Machiavellianism—accounted for the correlation between the motive of competition and self-reported use of aggressive humor. A comparison of the fit indices of our proposed and alternative models suggests that the effect flowed from the motive of competition to psychopathy and then to self-reported use of aggressive humor. Finally, these findings were held in both female and male respondents.

### 4.1. Did contests or mate-choice select for the use of aggressive humor?

This research adds to prior work on the use of aggressive humor by providing evidence that differentiates the Contests Hypothesis of the use of aggressive humor from the Mate-Choice Hypothesis. Prior research on aggressive humor under the framework of evolutionary psychology (Bressler and Balshine, 2006; Bressler et al., 2006; Greengross and Miller, 2008, 2011; Wilbur and Campbell, 2011; Cowan and Little, 2013; DiDonato et al., 2013; Zeigler-Hill et al., 2013; Hone et al., 2015; Ross and Hall, 2020) almost exclusively focused on the effect of using aggressive humor on users’ perceived desirability as a long- or short-term mate and mating success. However, those findings do not support either the Contests Hypothesis or the Mate-Choice Hypothesis. This is because traits selected by contests could also cause strong mate preferences.

For example, male muscularity is a sexually selected trait that facilitates mating competition (Puts, 2010), but it has a strong impact on women’s perceptions of men’s physical attractiveness (Sell et al., 2017) and positively correlates with men’s short-term mating success (Lassek and Gaulin, 2009). Relatedly, Brown et al. (2022) found that men who are more physically formidable are generally perceived to be more likely to use aggressive humor. However, because stronger men are perceived to be more attractive (Sell et al., 2017), to provide stronger support for the Contests Hypothesis would entail ruling out the potential confounding effect of perceived attractiveness.

The present research bypassed those problems by examining whether the motive of competition or that of courtship correlates more strongly with self-reported use of aggressive humor. Our results suggest that men and women primarily use aggressive humor—a type of verbal aggression and behavioral manifestation of being callous and reckless—to compete with mate rivals rather than to attract mates. At the same time, there is even a chance that people are less likely to use aggressive humor when the motive of courtship is chronically accessible. Given these findings, it appears that contests compared to mate choice are the main mechanism that selected for the psychological mechanism(s) producing the behavior of using aggressive humor. Indeed, other mechanisms of sexual selection such as mate choice would have little room to operate when organisms are able to repel competitors with physical force or threats of physical force (Puts, 2010).

### 4.2. Is the use of aggressive humor adaptative?

Because the use of aggressive humor hurts others and the self (see section “1.1 Costs of using aggressive humor”), many researchers considered aggressive humor as “a bad sense of humor” by being “negative,” “undesirable,” “maladaptive,” and “serving negative social functions” (DiDonato et al., 2013, p. 377; Zeigler-Hill et al., 2013, p. 204; Cann and Matson, 2014, p. 176; Cann et al., 2016, p. 259). These comments had likely not distinguished between ultimate- (i.e., functional) and proximate-level (i.e., mechanism) analyses (Scott-Phillips et al., 2011). We do not dispute that, at the proximate level, the use of aggressive humor correlates with many personality traits and interpersonal outcomes that would undermine users’ welfare measured in fitness units. However, this does not negate the possibility that the use of aggressive humor has promoted users’ reproduction in many other domains, that is, the behavior in question helps implement sexually and naturally selected functions. We provided evidence for one such fitness-enhancing effect (i.e., to facilitate mating competition), and other functions of the use of aggressive humor are possible, including settling intergroup conflicts (Hodson and MacInnis, 2016). Thus, future studies should distinguish between the ultimate and proximate levels of analysis when characterizing the effects of using aggressive humor.

### 4.3. The role of the dark triad personality traits on intrasexual competition

This research provides further evidence that Machiavellianism and especially psychopathy facilitates contests competition and motivates spiteful behaviors such as the use of aggressive humor (Carter et al., 2014; Goncalves and Campbell, 2014; Lyons et al., 2019). In particular, our model comparison (Figures 3A, C) suggests that, at least in the context of this research, the chronic accessibility of the motive of competition prescribes the behavioral tendencies labeled as psychopathy instead of the other way around. This finding is consistent with an adaptationist approach to human personalities (Lukaszewski et al., 2020) and helps reveal the evolved psychological mechanism(s) (e.g., negative fitness interdependence) that generate psychopathy.

Inconsistent with Lyons et al. (2019), we found the motive of competition positively and significantly correlated with narcissism before and after controlling for Machiavellianism and psychopathy. A possible cause for this difference is that Lyons et al. (2019) used Buunk and Fisher’s (2009) measure of competition whereas we used Chen and Chang’s (2015), albeit those two scales are semantically similar. Consistent with Kjaervik and Bushman (2021), we found a significant positive zero-order correlation between narcissism and self-reported use of aggressive humor, but this correlation dropped to being non-significant after we controlled for Machiavellianism and psychopathy. This finding highlights the importance of statistically removing the covariances between narcissism and the other two “dark” personality traits in predicting outcome variables of interest.

At the same time, this research adds to the body of work that clarifies the evolved function of sexually selected traits. This includes whether contests or mate choice has selected for physical aggression (Chen and Chang, 2015), male voice pitch (Puts and Aung, 2019), facial masculinity (Puts, 2010), and male muscularity (Lassek and Gaulin, 2009). It appears that those traits, now including the use of aggressive humor, are primarily selected by contests as weaponry traits.

This research also adds to the growing literature on female intrasexual competition. Prior research on female intrasexual competition mostly focused on the use of indirect aggression (e.g., gossiping, social exclusion) (Vaillancourt, 2013; Davis et al., 2018; Hess and Hagen, 2021) and coalitional physical aggression (Campbell, 2013). This research found that, while female respondents were generally less likely than male respondents to use aggressive humor, female respondents who were more chronically motivated to engage in competition with same-sex mate rivals tended to report stronger tendencies to use aggressive humor. This finding suggests that, in addition to indirect tactics and coalitional means, the use of aggressive humor—a type of direct aggression (e.g., teasing others in face-to-face interactions)—is also a tactic that women use to compete for mates.

### 4.4. Limitations and future directions

Comparing fit indices does not constitute direct evidence for causality, and our study design does not provide such evidence. Thus, future research may consider using randomized-controlled experiments or collecting longitudinal data to stringently test the Contests Hypothesis of the use of aggressive humor. As a second limitation, we studied respondents’ self-reported tendency to use aggressive humor, while a more proper test of the Contests Hypothesis would be to use behavioral data. This is because evolved psychological programs increase individuals’ fitness by producing environmentally appropriate behaviors. Thus, we call for studying aggressive humor with naturalistic studies or lab experiments that simulate scenarios of intrasexual competition. As a third limitation, while we argued and provided evidence that the use of aggressive humor mainly correlates with the motive of competition, the measurement we used tapped into the propensity to use aggressive humor as a trait instead of measuring the use of aggressive humor as a situational tactic. Measuring the trait-level use of aggressive humor suited our purpose because we were interested in the evolved mechanism producing this spiteful behavior. However, future research may consider adapting the HSQ to measure the situational use of aggressive humor.

Lastly, we examined the motivational antecedents of self-reported use of aggressive humor but not its outcomes. If aggressive humor is a form of verbal aggression signaling hostility, it should elicit stronger retaliation from rivals higher on threat potential (e.g., trait aggressiveness, physical strength) than from rivals with lower threat potential (Zhang et al., 2021). Further, if the Contests Hypothesis is correct, men’s use of aggressive humor should lead to mating success through increases in perceived dominance but not prestige.

As one possibility, the tendency to use aggressive humor as a relatively stable personality trait (Martin et al., 2003) would predispose one to use offensive jokes to compete for social status. People relatively high on the tendency to use aggressive humor may or may not have a specific target individual in mind at a given moment. However, prior research (Huo et al., 2012) suggests that they would rarely tease a superior but mostly direct offensive jokes toward individuals of lower ranks (to maintain the hierarchy) and peers (to jockey for a higher position by seeing who accepts the tease and who retorts).

As another possibility, a person could be generally pleasant but deploys aggressive humor as a situational tactic to avenge someone who just insulted him. In this case, the use of aggressive humor helps one protect his “honor,” thereby attracting or retaining mates (Shackelford, 2005). In either case, the use of aggressive humor enables the aggressor to have plausible deniability (e.g., “Take it easy. I’m only teasing!”) in an attempt to hurt a target individual and may thus be less costly (e.g., avoiding quick escalation and social disapproval) than using outright physical aggression in early stages of an antagonistic interaction. Importantly, whether the use of aggressive humor is a personality trait or a situational tactic, it—similar to trait hostility, Machiavellianism, psychopathy, and various types of aggression—is unlikely designed to increase one’s likeability but to hurt people whose welfare negatively relates to the self. The net benefits accrued by hurting an “enemy” (e.g., mating success despite being disliked by peers) are likely what has maintained such a spiteful behavior as using aggressive humor.

## Data availability statement

The data and replication R script are hosted here: https://osf.io/yabtk/?view_only=4c5b25d5462c4018937604ba5a70b218.

## Ethics statement

The studies involving human participants were reviewed and approved by the University of Hawai‘i at Mānoa Institutional Review Board. The patients/participants provided their written informed consent to participate in this study.

## Author contributions

BD conceived and designed the study. BD and JZ collected and analyzed the data and wrote the manuscript. Both authors contributed to the article and approved the submitted version.

## Footnotes

1 A HIT is a task that a registered Amazon Turk user may work on and collect reward for.
