# Supplementary material for: The motive of competition but not courtship positively correlates with self-reported use of aggressive humor: A critical test of the contests- vs. mate-choice hypotheses
Source: Front Psychol. 2023 Jan 11;13:1056217. doi: 10.3389/fpsyg.2022.1056217 (PMC9874086; doi:10.3389/fpsyg.2022.1056217)
Supplement: Supplementary file 1 [file Table_1.docx]

**Supplemental Information**

To

The Motive of Competition but not Courtship Positively Correlates with Self-Reported Use of Aggressive Humor: A Critical Test of the Contests- versus Mate-Choice Hypotheses

**Table S1**

*Predicting Self-Reported Use of HumorCorrelations from Motives of Competition and Courtship Controlling for Age*

| Parameter | ß | *SE* | *Z* | *p* |
| --- | --- | --- | --- | --- |
| Predicting aggressive humor |  |  |  |  |
| Competition | 0.36 | 0.05 | 8.03 | < .001 |
| Courtship | -0.10 | 0.05 | -1.95 | .05 |
| Age | -0.19 | 0.05 | -4.08 | < .001 |
| Predicting affiliative humor |  |  |  |  |
| Competition | -0.33 | 0.05 | -6.06 | < .001 |
| Courtship | 0.19 | 0.06 | 3.19 | .001 |
| Age | -0.10 | 0.05 | -2.05 | .04 |
| Predicting self-enhancing humor |  |  |  |  |
| Competition | -0.09 | 0.06 | -1.55 | .12 |
| Courtship | 0.21 | 0.06 | 3.30 | .001 |
| Age | -0.01 | 0.05 | -0.25 | .80 |
| Predicting self-defeating humor |  |  |  |  |
| Competition | 0.32 | 0.05 | 6.54 | < .001 |
| Courtship | 0.16 | 0.05 | 2.97 | .003 |
| Age | -0.10 | 0.05 | -2.15 | .03 |

**Table S2**

*Sex Differences in Self-Reported Use of Humor*

|  | Men | Women | *t* | *df* | *p* | Cohen’s *d* |
| --- | --- | --- | --- | --- | --- | --- |
| Affiliative humor | 5.23  (1.15) | 5.17  (1.20) | 0.31 | 434.2 | .75 | 0.05 |
| Self-enhancing humor | 4.85  (1.06) | 4.72  (1.25) | 0.85 | 419.2 | .40 | 0.11 |
| Aggressive humor | 3.70  (1.06) | 3.07  (1.03) | 6.36 | 437.0 | < .001 | 0.53 |
| Self-defeating humor | 3.77  (1.14) | 3.47  (1.27) | 2.62 | 428.5 | .009 | 0.25 |

*Note*: Standard deviations are in parentheses. Degrees of freedom have decimals because we used Welch’s *t*-tests that are robust to unequal variances. Means and standard deviations were based on original variables. Welch’s t-tests were based on Z-transformed variables corrected for skew (see main text for details).

**Table S3**

*Testing the Indirect Effects of Machiavellianism (Model 1) and Psychopathy (Model 2)Controlling for the Self-Reported Use of Affiliative, Self-Enhancing, and Self-Defeating Humor*

|  | Model 1 | Model 2 |
| --- | --- | --- |
| Parameter |  |  |
| Predicting Machiavellianism |  |  |
| Competition | 0.61***  (.03) |  |
| Predicting psychopathy |  |  |
| Competition |  | 0.70***  (.03) |
| Predicting aggressive humor |  |  |
| Competition | 0.05  (.06) | -0.03  (.06) |
| Machiavellianism | 0.39***  (.05) |  |
| Psychopathy |  | 0.52***  (.06) |
| Affiliative humor | 0.13**  (.05) | 0.22***  (.05) |
| Self-enhancing humor | 0.05  (.05) | -0.01  (.05) |
| Self-defeating humor | 0.26***  (.05) | 0.19***  (.05) |

*Note*:Standard errors are in parentheses. *** *p*< .001, ** *p*< .01.

**Table S4**

*PredictingSelf-Reported Use of Humor from the Motives of Competition and Courtship and in Female Respondents*

| Parameter | ß | *SE* | *Z* | *p* |
| --- | --- | --- | --- | --- |
| Predicting aggressive humor |  |  |  |  |
| Competition | 0.48 | 0.06 | 8.28 | < .001 |
| Courtship | -0.15 | 0.08 | -1.90 | .06 |
| Predicting affiliative humor |  |  |  |  |
| Competition | -0.29 | 0.08 | -3.50 | < .001 |
| Courtship | 0.15 | 0.09 | 1.68 | .09 |
| Predicting self-enhancing humor |  |  |  |  |
| Competition | -0.06 | 0.09 | -0.62 | .54 |
| Courtship | 0.17 | 0.11 | 1.58 | .11 |
| Predicting self-defeating humor |  |  |  |  |
| Competition | 0.34 | 0.07 | 4.98 | < .001 |
| Courtship | 0.17 | 0.08 | 2.15 | .03 |

*Note*: *** *p*< .001, ** *p*< .01.

**Table S5**

*PredictingSelf-Reported Use of Humor from the Motives of Competition and Courtship in Male Respondents*

| Parameter | ß | *SE* | *Z* | *p* |
| --- | --- | --- | --- | --- |
| Predicting aggressive humor |  |  |  |  |
| Competition | 0.36 | 0.06 | 6.51 | < .001 |
| Courtship | -0.18 | 0.07 | -2.52 | .01 |
| Predicting affiliative humor |  |  |  |  |
| Competition | -0.31 | 0.07 | -4.65 | < .001 |
| Courtship | 0.25 | 0.08 | 3.00 | .003 |
| Predicting self-enhancing humor |  |  |  |  |
| Competition | -0.10 | 0.06 | -1.51 | .13 |
| Courtship | 0.25 | 0.08 | 3.24 | .001 |
| Predicting self-defeating humor |  |  |  |  |
| Competition | 0.35 | 0.06 | 5.59 | < .001 |
| Courtship | 0.15 | 0.08 | 1.90 | .06 |

*Note*: *** *p*< .001, ** *p*< .01.

**Table S6**

*Predicting Self-Reported Use of Aggressive Humorfrom the Motive of Competition with Dark Triad Personalities as Mediators in Female Respondents*

| Parameter | ß | *SE* | *Z* | *p* |
| --- | --- | --- | --- | --- |
| Predicting Machiavellianism |  |  |  |  |
| Competition | 0.62 | 0.04 | 13.8 | < .001 |
| Predicting psychopathy |  |  |  |  |
| Competition | 0.67 | 0.05 | 13.9 | < .001 |
| Predicting narcissism |  |  |  |  |
| Competition | 0.59 | 0.04 | 13.3 | < .001 |
| Predicting aggressive humor |  |  |  |  |
| Competition | 0.00 | 0.08 | 0.02 | .98 |
| Machiavellianism | 0.21 | 0.08 | 2.85 | .004 |
| Psychopathy | 0.48 | 0.08 | 5.85 | < .001 |
| Narcissism | -0.11 | 0.08 | -1.42 | .16 |

**Table S7**

*Predicting Self-Reported Use of Aggressive Humor from the Motive of Competition with Dark Triad Personalities as Mediators in Male Respondents*

| Parameter | ß | *SE* | *Z* | *p* |
| --- | --- | --- | --- | --- |
| Predicting Machiavellianism |  |  |  |  |
| Competition | 0.57 | 0.05 | 12.8 | < .001 |
| Predicting psychopathy |  |  |  |  |
| Competition | 0.67 | 0.04 | 16.4 | < .001 |
| Predicting narcissism |  |  |  |  |
| Competition | 0.54 | 0.05 | 12.1 | < .001 |
| Predicting aggressive humor |  |  |  |  |
| Competition | -0.11 | 0.10 | -1.10 | .27 |
| Machiavellianism | 0.33 | 0.09 | 3.66 | < .001 |
| Psychopathy | 0.32 | 0.10 | 3.07 | .002 |
| Narcissism | -0.00 | 0.08 | -0.02 | .98 |

**Table S8**

*Testing the Indirect Effects of Machiavellianism (Model 1) and Psychopathy (Model 2)in Female Respondents*

|  | Model 1 | Model 2 |
| --- | --- | --- |
| Parameter |  |  |
| Predicting Machiavellianism |  |  |
| Competition | 0.62***  (.04) |  |
| Predicting psychopathy |  |  |
| Competition |  | 0.67***  (.05) |
| Predicting aggressive humor |  |  |
| Competition | 0.18**  (.07) | 0.03  (.08) |
| Machiavellianism | 0.34***  (.07) |  |
| Psychopathy |  | 0.55***  (.08) |

*Note*: Standard errors are in parentheses. *** *p*< .001, ** *p*< .01.

**Table S9**

*Testing the Indirect Effects of Machiavellianism (Model 1) and Psychopathy (Model 2)in Male Respondents*

|  | Model 1 | Model 2 |
| --- | --- | --- |
| Parameter |  |  |
| Predicting Machiavellianism |  |  |
| Competition | 0.57***  (.05) |  |
| Predicting psychopathy |  |  |
| Competition |  | 0.67***  (.04) |
| Predicting aggressive humor |  |  |
| Competition | 0.4  (.07) | -0.01  (.09) |
| Machiavellianism | 0.44***  (.09) |  |
| Psychopathy |  | 0.44***  (.10) |

*Note*: Standard errors are in parentheses. *** *p*< .001, ** *p*< .01.

**Figure S1**

*Comparing Model Fit of Three Competing Mediation Models in Female Respondents*


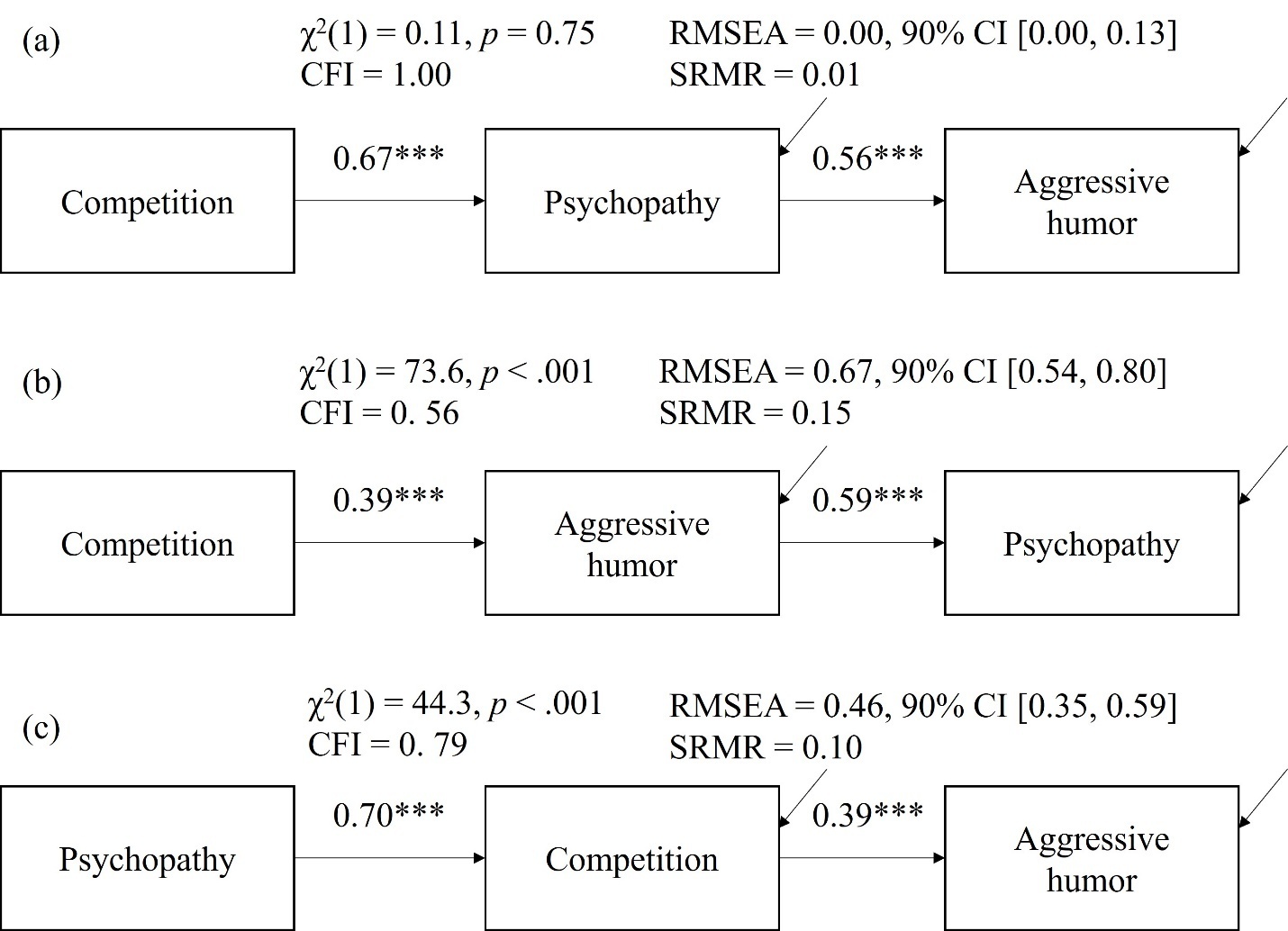


*Note*: Arrow lines pointing toward the mediator- and outcome variables represent residual terms. Larger *p*-values of the chi-square test, larger CFI values, smaller RMSEA values, and smaller SRMR values indicate better model fit. *** *p*< .001

**Figure S2**

*Comparing Model Fit of Three Competing Mediation Models in Male Respondents*


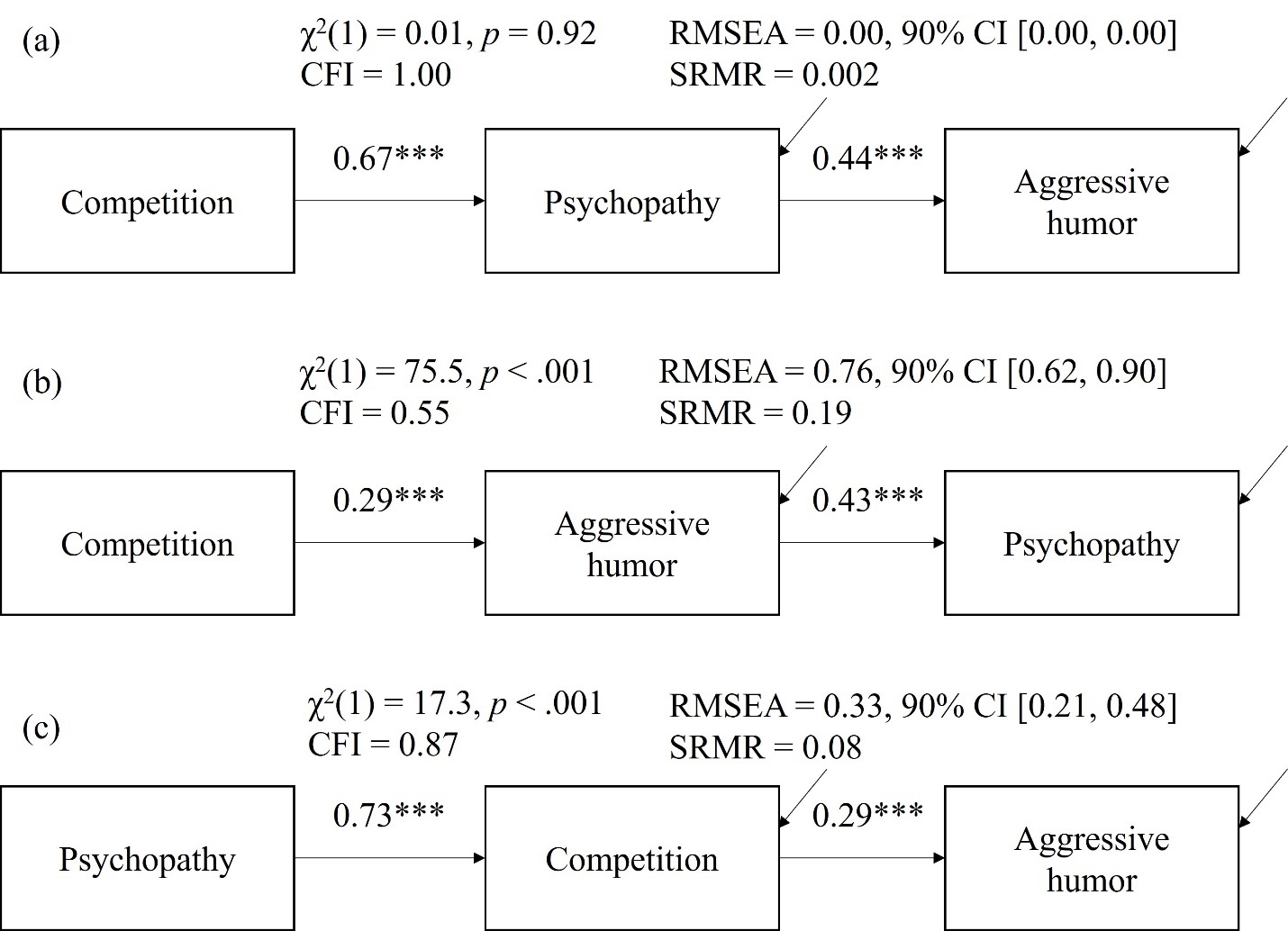


*Note*: Arrow lines pointing toward the mediator- and outcome variables represent residual terms. Larger *p*-values of the chi-square test, larger CFI values, smaller RMSEA values, and smaller SRMR values indicate better model fit. *** *p*< .001
